# Association between emergency physician self-reported empathy and patient satisfaction

**DOI:** 10.1371/journal.pone.0204113

**Published:** 2018-09-13

**Authors:** Hao Wang, Jeffrey A. Kline, Bradford E. Jackson, Jessica Laureano-Phillips, Richard D. Robinson, Chad D. Cowden, James P. d’Etienne, Steven E. Arze, Nestor R. Zenarosa

**Affiliations:** 1 Department of Emergency Medicine, Integrative Emergency Services, John Peter Smith Health Network, Fort Worth, TX, United States of America; 2 Department of Emergency Medicine, University of Indiana School of Medicine, Indianapolis, IN, United States of America; 3 Center for Outcomes Research, John Peter Smith Health Network, and University of North Texas Health Science Center, School of Public Health, Fort Worth, TX, United States of America; 4 Office of Clinical Research, John Peter Smith Health Network, Fort Worth, TX, United States of America; University of Minho, PORTUGAL

## Abstract

**Background:**

Higher physician self-reported empathy has been associated with higher overall patient satisfaction. However, more evidence-based research is needed to determine such association in an emergent care setting.

**Objective:**

To evaluate the association between physician self-reported empathy and after-care instant patient-to-provider satisfaction among Emergency Department (ED) healthcare providers with varying years of medical practice experience.

**Research design:**

A prospective observational study conducted in a tertiary care hospital ED.

**Methods:**

Forty-one providers interacted with 1,308 patients across 1,572 encounters from July 1 through October 31, 2016. The Jefferson Scale of Empathy (JSE) was used to assess provider empathy. An after-care instant patient satisfaction survey, with questionnaires regarding patient-to-provider satisfaction specifically, was conducted prior to the patient moving out of the ED. The relation between physician empathy and patient satisfaction was estimated using risk ratios (RR) and their corresponding 95% confidence limits (CL) from log-binomial regression models.

**Results:**

Emergency Medicine (EM) residents had the lowest JSE scores (median 111; interquartile range [IQR]: 107–122) and senior physicians had the highest scores (median 119.5; IQR: 111–129). Similarly, EM residents had the lowest percentage of “very satisfied” responses (65%) and senior physicians had the highest reported percentage of “very satisfied” responses (69%). There was a modest positive association between JSE and satisfaction (RR = 1.04; 95% CL: 1.00, 1.07).

**Conclusion:**

This study provides evidence of a positive association between ED provider self-reported empathy and after-care instant patient-to-provider satisfaction. Overall higher empathy scores were associated with higher patient satisfaction, though minor heterogeneity occurred between different provider characteristics.

## Introduction

Empathy plays a critical role in rapport building between healthcare providers and patients [1]. Higher physician empathy scores are associated with higher patient satisfaction, better patient clinical outcomes, and fewer medical litigation actions [2,3]. Various instruments are available for measuring provider empathy including Jefferson Scale of Empathy (JSE) and Jefferson Scale of Patient Perception of Physician Empathy (JSPPPE) [4–7]. Comparison studies on the JSE and JSPPPE to determine provider empathy have been done before and their results are inconsistent [8,9]. One family medicine study showed high correlation between JSE and JSPPPE to determine provider empathy [8], whereas other studies failed to duplicate such findings [9–11]. The controversial findings might be due to the biased patient perception of provider empathy in comparison to provider self-assessed since empathy determination requires certain time to develop and might be skewed due to limited time within a single patient encounter [7]. This could particularly be occurring in stressful environments where there is a lack of relationship built between patients and providers on empathic understanding [9,12]. The JSE was initially derived and used for empathy evaluations of health professions students and proved to be valid in terms of its construction, internal consistency, prediction, and test-retest reliabilities [8,13–15]. The current, expanded version is used to measure empathy among all healthcare providers including attending physicians, resident physicians, nurses, advanced practice providers, and pharmacists [16–18]. Of note, the JSE has been found to vary among providers depending upon medical practice, gender and experience [15] [19,20] [21–23]. However, it has been found to be stable, with high test-retest reliabilities [13,24].

Patient satisfaction has been associated with provider empathy in previous studies. Higher patient perception of physician empathy scores have been associated with higher patient satisfaction in a primary care setting [14] and higher provider self-reported empathy has also been observed with better patient satisfaction among otolaryngology residents [25]. However, such findings have not been reported and might not be duplicable in the emergency care setting. We assume that emergency care setting is different than other clinical setting with the fact of both ED patients and providers might experience more stress [26], such unique characteristic might alter the association between provider empathy and patient satisfaction. Previous studies showed that patients with higher anxiety levels correlated with lower patient satisfaction [27] and providers with better stress control tended to have higher patient satisfactions [28]. Given that empathy could be affected by stress and anxiety [29,30], it is important to determine the association between physician empathy and patient satisfaction in the ED. Accounting for the short duration rendered between physicians and patients in the emergency care setting with potential biased perception of physician empathy determined by the patients, we considered using self-reported JSE for physician empathy assessment in this study.

Therefore, the aim of this study was to explore the association between Emergency physician self-reported empathy and after-care instant patient satisfaction within a prospective cohort. Moreover, we sought to assess the heterogeneity of this relationship across provider gender and level of professional training and experience.

## Methods

### Study design and population

This was a prospective observational study conducted from July 1 through October 31, 2016. The study hospital is an urban tertiary care, Level 1 trauma center, chest pain center, and stroke center. The study ED experiences more than 120,000 annual visits. All patients that presented to the ED during the study time frame were eligible for enrollment. Upon disposition, data were collected for after-care instant patient satisfaction surveys (S1 Table). The JSE survey was conducted at the study ED and was administered to attending physicians and EM residents with varying levels of experience in July 2016. We included all ED providers whose JSE surveys were completed and matched to all patients who had completed their after-care instant patient satisfaction survey. The data were collected from two sources, the ED providers as well as patients who presented to the ED during the study period. Data were excluded for: (1) providers who did not participate in the JSE survey; (2) patients who did not participate in the satisfaction survey for any reason; (3) patients whose surveyors entered incorrect information (e.g., answers not chosen from pre-determined options [menu] or answers unrelated to the questions); (4) patients who completed less than 20% of the survey; and (5) patients who did not identify their providers (attending physicians and/or residents) when completing the survey. This study was approved by the John Peter Smith Health Network Institutional Review Board with waived consent.

### Provider empathy

The primary explanatory variable of interest in this study was provider empathy as measured using the JSE. In brief, the current edition of the JSE is a revised version of the scale for physicians and health professionals (Health Professional Version or HP-version) [15]. It includes a 20-item questionnaire with positively and negatively worded questions. Responses to each of the positive questions is on a 7-point Likert Scale (“strongly disagree” = 1 to “strongly agree” = 7). The negative questions are reverse scored using the same 7-point Likert Scale (“strongly disagree” = 7 to “strongly agree” = 1). Taking into account the reverse scored questions, the grand total is summed across all 20 questions for scores ranging from 20 to 140. Considering short duration of the study, providers were asked to complete the JSE only once at the start of the study.

### Patient satisfaction survey

The study’s primary outcome of interest was patient reported satisfaction with the treating ED providers who completed the JSE. An after-care instant patient satisfaction survey (Qualitick, Clearwater, FL) was conducted at the end of the ED encounter, prior to patient moving out of ED (S1 Table). Specifically, patients were asked to rate their satisfaction with providers using five different categories: “very dissatisfied”; “dissatisfied”; “neither satisfied nor dissatisfied”; “satisfied”; and “very satisfied.” Patient surveys included treating provider name(s) (attending and/or resident). Satisfaction was assessed separately for attending physicians and residents regardless whether the patient was seen by both or just one. A computer tablet housing the confidential survey was provided to patients and/or their designees in a private setting, away from healthcare providers. The identification of patients participating the survey was optional and patients who completed the survey could remain anonymous. The expected time for survey completion was less than five minutes. The study institution treats a large percentage of Spanish speaking patients; in order to preempt a sizeable amount of Spanish-speaking patients, Spanish version of patient satisfaction survey were prepared and provided to participants. Unlike other measures of patient reported satisfaction, which are assessed at later dates and are more subject to the influence of recall and other biases, after-care instant assessments provide a better measure of specific provider interactions.

### Variables

Experience level was classified across both physicians and residents for an overall categorization scheme with 3 strata: resident (post graduate year [PGY]-1, PGY-2, and PGY-3), junior physician (< 5 years post-graduate experience), and senior physician (≥ 5 years post-graduate experience). Other provider variables of interest included sex and race/ethnicity. Patient characteristics included age, sex, and race/ethnicity.

### Data analysis

The distribution of responses to patient-to-provider satisfaction was left skewed, with responses being predominantly (> 94%) classified as “very satisfied” and “satisfied.” Therefore, we dichotomized the outcome as “very satisfied” and “not very satisfied” (i.e. “satisfied”, “neither satisfied nor dissatisfied”, “dissatisfied”, and “very dissatisfied”). In order to estimate the association between empathy and patient-to-provider satisfaction, we made the following assumptions. Given that multiple patients may have been seen by different ED providers and were assessed separately, we treated each patient-provider interaction independently. In addition, we assumed that each interaction with providers was independent from one another, and that patients who presented to the ED should be considered independent of their previous visit. Under these assumptions we aimed to reduce the influence of correlation between providers treating the same patients and within repeated patient visits. These assumptions were supported by the low intraclass correlation (ICC = 0.039) estimated from a 2-level model where patients were nested within providers. The ICC suggested that approximately 4% of the variance of the outcome was explained by between subject variability. Given the low ICC, model parsimony, and the clinical assumptions of each patient-provider interaction being independent, we proceeded without accounting for a hierarchical structure. Subsequently, we estimated risk ratios (RR) and their corresponding 95% confidence limits (CL) using generalized linear models with a log link function and binomial distribution[31,32]. This approach is appropriate for estimating risk ratios from binary outcomes while adjusting for confounders of the exposure-relation when the prevalence of the outcome is not rare (i.e. >10%). In the present study, 67% of responses were “very satisfied”, thus exceeding the suggested threshold of 10% for rare outcomes. To present a clinically meaningful interpretation of the JSE association, RRs are presented as a 10-unit increase in overall JSE score. For all observations, JSE scores were centered around the overall sample mean score and divided by 10. This approach provides an anchored unit increase as opposed to a data-based value (e.g. standard deviation) which facilitates future studies reference comparisons. We estimated the overall provider, sex-specific, and experience-stratified associations between the overall empathy score and patient satisfaction with adjustment for a minimally sufficient set of covariates to reduce confounding bias [33]. In the log-binomial model for the overall sample association, the covariates identified using a directed acyclic graph were provider gender and experience level [34]. The sex-specific associations were estimated adjusting for physician level of experience, and the provider experience-stratified estimates adjusted for provider gender. Analyses were performed using Stata v14.0 (College Station, TX) and SAS v9.4 (Cary, NC).

## Results

From July 1, 2016 through October 31, 2016, 66 providers were asked to complete the JSE. Forty-one (62%) of these ED providers completed the JSE surveys. All JSE surveys contained complete data and were supplied by 18 faculty and 23 residents. These physicians cared for 1,238 unique patients who completed after-care instant satisfaction surveys. Emergency department staff asked a total of 2,447 patients to complete the patient satisfaction survey at disposition, and only 10 (0.41%) did not complete the survey. There were 1,139 (47%) observations who were excluded because they were not treated by an ED provider who completed a JSE survey, yielding a total of 1,308 patient satisfaction surveys matched to the study ED providers. Moreover, 70 observations were from patients with multiple visits. Based on our assumptions of independence, the analysis dataset comprised 1,572 patient-provider interactions between 41 providers and 1,238 patients during the study period.

The general study provider and patient information is detailed in Table 1. Briefly, ED providers were predominantly male (71%), Caucasian (78%) with roughly equivalent numbers of participants across all groups (PGY-1, PGY-2, PGY-3, junior, and senior physicians). The overall provider median JSE score was 114 (IQR: 109–121). Surveyed patients were, on average, 45 years of age, equally female (49%), comprising mostly non-Hispanic White (37%) and non-Hispanic Black (31%) race/ethnicity. Generally, patients were predominantly “very satisfied” (67%) or “satisfied” (32%) with their ED provider. The overall point estimate for the association between JSE score and patient satisfaction for a 10-unit increase in JSE was 1.04 (95% CL: 1.00, 1.07).

**Table 1:** Characteristics of emergency department health providers, enrolled patient population, and patient satisfaction.

**ED Health Provider General Information **		**N = 41**	**%**
Provider Experience			
	Senior Attending Physicians	8	19.5
	Junior Attending Physicians	10	24.4
	Emergency Medicine Residents		
	PGY-1	10	24.4
	PGY-2	6	14.6
	PGY-3	7	17.1
Sex	Male	29	70.7
	Female	12	29.3
Race / Ethnicity	Asian	8	19.5
	African American	1	2.4
	Caucasian	21	78.1
JSE	Median (IQR)	114 (109, 121)	
	Mean (SD)	113.2 (12.5)	
	Minimum, Maximum	80, 134	
**Patient General Information and Patient Satisfaction**		**N**	**%**
Number of Patient-Provider Interactions		1572	
Patient Satisfaction across Encounter			
Very satisfied		1047	66.6
Not very satisfied		525	33.4
Number of Unique Patients Enrolled		1238	
Age (years)	Median (IQR)	47 (33, 57)	
	Mean (SD)	45.3 (16.4)	
Sex	Male	634	51.2
	Female	603	48.8
Race / Ethnicity	Hispanic	323	26.1
	Non-Hispanic Black	388	31.3
	Non-Hispanic Other	60	4.9
	Non-Hispanic White	467	37.7
**Overall Association between JSE and Patient Satisfaction**		RR, 95% CL	
	10-unit JSE Increase	1.04 (1.00, 1.07)	

Abbreviations: n, Number; ED, Emergency Department; PGY, Post-graduate Year; JSE, Jefferson Scale of Empathy; SD, Standard Deviation; IQR, Interquartile Range; RR, Risk Ratio; CL, Confidence Limit

Table 2 presents both overall and stratum-specific distributions of provider satisfaction and the risk ratios for “very satisfied” versus “not very satisfied” across all patient-provider interactions. Male ED providers had a slightly lower proportion of high satisfaction scores than females (males = 66% versus females = 69%). The distributions of JSE scores were similar between male and female ED providers. For a 10-point increase in JSE score, we found that both male and female providers had a positive association, where the estimate for males (RR = 1.04; 95% CL: 1.01, 1.08) was slightly higher and with less variability than females (RR = 1.02; 95% CL: 0.88, 1.17).

**Table 2:** Stratum specific distributions of patient satisfaction and jefferson scale of physician empathy.

	Provider Sex		Provider Practice Experience		
	Male	Female	EM Resident	Junior Physician	Senior Physician
**Patient Satisfaction with ED Provider**^**a**^ **(n, %)**	1203	369	769	491	312
Very Satisfied	793 (66%)	254 (69%)	501 (65%)	330 (67%)	216 (69%)
Satisfied	391 (33%)	112, (30%)	259 (34%)	152 (31%)	92 (30%)
Neither / Nor	17 (1%)	1 (0.27%)	6 (0.78%)	9 (2%)	3 (0.96%)
Dissatisfied	1 (0.08%)	1 (0.27%)	1 (0.13%)	0 (0%)	1 (0.32%)
Very Dissatisfied	1 (0.08%)	1 (0.27%)	2 (0.26%)	0 (0%)	0 (0%)
**JSE (n)**	29	12	23	10	8
Median, IQR	114, (106, 122)	114 (111, 118)	111, (107, 122)	113 (109, 117)	120, (111, 129)
Mean (SD)	112.2 (13.9)	115.8 (7.9)	112.2 (11.9)	111.6 (13.3)	118.1 (13.5)
Minimum, Maximum	80, 134	106, 132	81, 130	80, 132	93, 134
**Measure of Association**					
RR (95% CL)^b^	1.04 (1.01, 1.08)	1.02 (0.88, 1.17)	1.00 (0.95, 1.05)	1.11 (1.04, 1.18)	1.02 (0.94, 1.11)

^a^Sample sizes are for the 1,572 patient-provider interactions across the 1,238 patients in the study with 264 represented more than once.

^b^Risk ratios and confidence limits are presented for a 10-unit increase in JSE score

Abbreviations: n, Number; EM, Emergency Medicine; ED, Emergency Department; JSE, Jefferson Scale of Physician Empathy; SD, Standard Deviation; IQR, Interquartile Range; RR, Risk Ratio; CL, Confidence Limit

**Fig 1:**
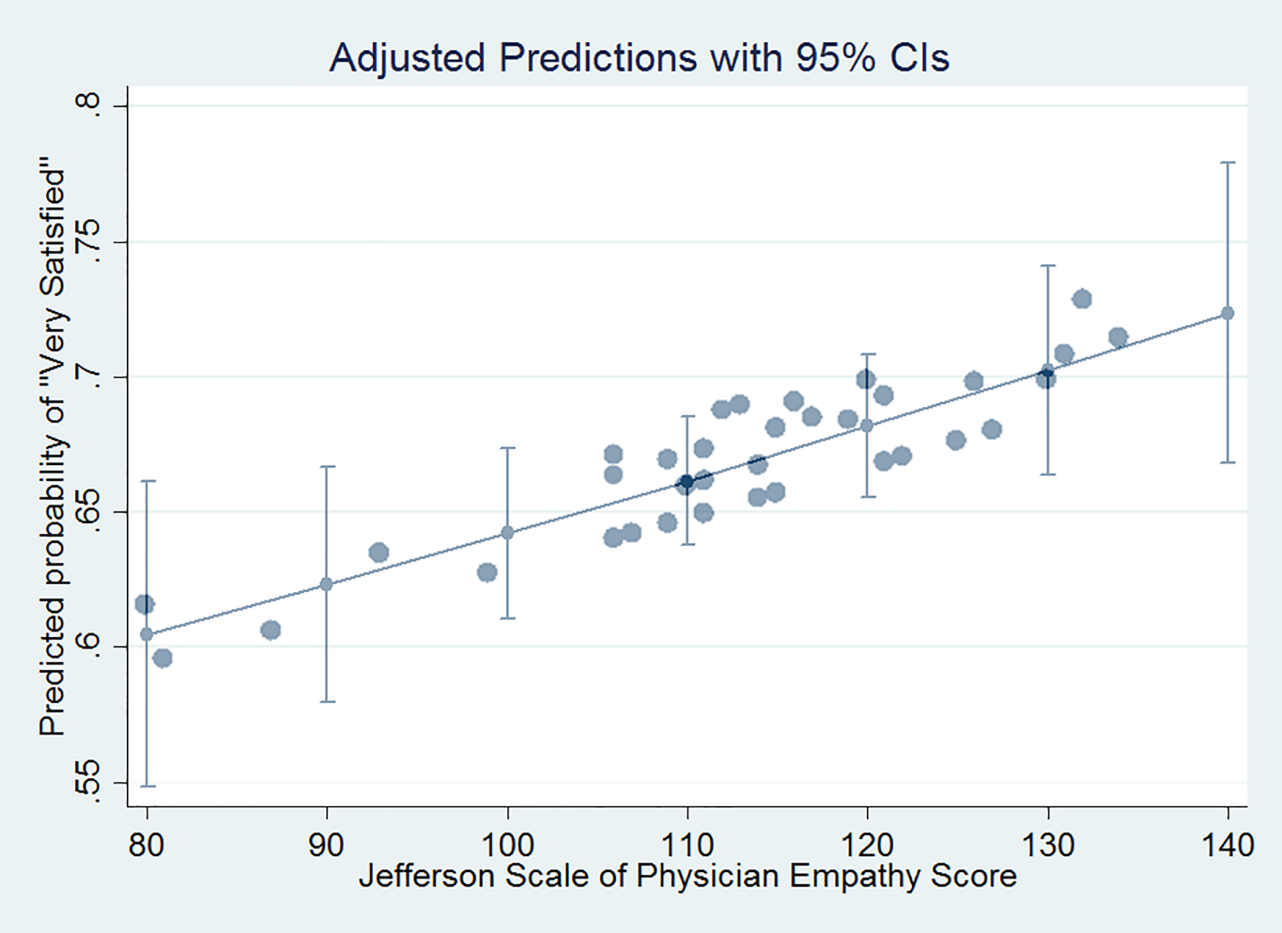
Shows association between JSE score and patient satisfactions.

Patient satisfaction with ED providers varied across levels of provider experience. While the majority of patients were “very satisfied” with providers, EM residents (PGY-1, PGY-2, and PGY-3 combined) had the lowest percentage of “very satisfied” responses (65%) and senior physicians had the highest reported percentage of “very satisfied” responses (69%). Similarly, there was heterogeneity across provider experience level in terms of the distribution of JSE score, where EM residents had the lowest scores (median 111; IQR: 107–122) and senior physicians had the highest scores (median 120 IQR: 111–129). There were positive associations between provider JSE score and patient-to-provider satisfaction; however, the magnitude of the association was greatest among junior physicians (RR = 1.11; 95% CL: 1.04, 1.18). For the other provider experience levels, the point estimates were close to 1.0 and the confidence intervals exhibited heterogeneity with increasing practice experience. Fig 1 presents the predicted probabilities of “very satisfied” from the log binomial model. It illustrates the overall positive patient satisfaction and JSE scores in our study’s ED provider populations.

## Discussion

Our results suggest that JSE scores are positively associated with after-care instant patient-to-provider satisfaction. Utilizing a direct pairing between instant ED provider-to-patient satisfaction surveys, our data suggest an approximately 5% relative increase in patients’ satisfaction with their provider for 10-point increases in the JSE. Our data support the study hypothesis that higher physician self-reported empathy score is concurrent with better patient satisfaction. We observed minor heterogeneity in the measure of association among providers of different sex and/or practice experience characteristics. The observed differences in the measures of association were predominantly in the positive direction, with the exception being among EM residents in which the point estimate indicated a null association. Our data add to existing literature by providing associations between JSE score and after-care instant patient-to-provider satisfaction in an extremely high volume and relatively busy, stressful environment. To the best of our knowledge, such association has not been reported in the literature.

We found minor subgroup heterogeneity, with relatively higher self-reported empathy scores among senior attending physicians compared to junior attendings and residents. Our results had opposite findings to previous reports that lower empathy scores are associated with increased years of clinical practice [35,36]. We attribute this association is due to an emphasis on faculty stewardship/training in patient-centered care. Over 80% of our senior attending physicians are members of ED leadership and take part in the study site’s daily ED operations. These senior attending physicians have (1) taken part in the AIDET (Acknowledge, Introduce, Duration, Explanation, and Thank) stewardship work-shop at the study ED, (2) participated in monthly ED operational and quality metric leadership meeting specifically addressing patient satisfaction and patient-centered cares, and (3) attended various hospital quality committees (e.g. sepsis, trauma, cardiac associated patient quality committees, etc.). We assume that such an environment fully dedicated to patient-centered care and patient safety provides department cultures with an emphasis on humanities, compassion, and quality which might promote positive provider empathy. On the other hand, another study reported a “V” shape association between compassionate attitudes and professional seniority in vascular surgeons and posited that senior surgeons were more prone to favor compassionate attitudes when facing clinical ethics [37]. Such changes might be attributed to more stress and overloaded work among junior providers [37]. Overall, we are unable to determine the direct cause of high empathy scores among senior attending physicians in our study but leave open the possibility of increased empathy with frequent exposure of advanced training and emphasizing humanities, compassion, and quality, which have been supported in other current literature [38–41]. Furthermore, our results suggest similar physician empathy scores regardless of provider gender which is inconsistent with other studies favoring higher scores in female providers [15,19]. This might be due to the relatively small sample size of female providers, especially very few female senior attending physicians (n = 3).

The findings of our study should be considered in light of several limitations. First, this was a prospective observational single-center study in which patients and providers were not randomly selected for participation. This convenience sample of study participants may lack generalizability and may potentially influence the observed exposure-outcome relationship. Second, the JSE was administered only once, and the providers’ perceptions may have fluctuated during the study. However, given the short duration of this study, we assumed such changes might be minimal. Third, the sample’s point estimates may be biased by a 40% nonresponse rate and the single center sample. Fourth, patient-to-provider satisfaction results were dichotomized into two categories (e.g. “very satisfied” versus “not-very-satisfied”) due to most of the surveys were categorized either “very satisfied” or “satisfied”. Such “ceiling effect” findings are not uncommon in similar study [9]. With extremely skewed data, the final result interpretations may be deviated and the true association between patient satisfaction and provider empathy might not be fully captured. The mean JSE scores and generally favorable satisfaction with providers may have resulted in our analytic presentation to that of a ceiling effect. Our regression model is centered about the mean score of 113 leaving limited room for improved JSE scores. To further explore this, we categorized the JES scores into quartiles, and estimated linear contrasts comparing the lowest quartile with higher quartiles and observed a trend toward a risk ratio point estimate of 1.0 moving up the quartiles (S2 Table). Fifth, patient satisfaction was not compared with traditional satisfaction surveys and other factors that might affect patient satisfaction with specific providers (e.g., patients with psychosocial risks, influence of pain medications, patients with different relative acuity levels or varying levels of disease severity). Lastly, though there were inconsistent findings reported in the literature regarding physician self-assessed empathy versus patient perceived physician empathy in related to patient satisfaction, the current study did not perform such comparisons. Therefore, a prospective multi-center study including diversity of patient populations, comparing different provider empathy measurement tools, and using various patient satisfaction evaluations with a larger sample size is warranted to externally validate our results.

In conclusion, our study provides evidence of a positive association between ED physician self-reported empathy scores and instant patient-to-provider satisfaction. Overall higher empathy scores are associated to higher patient satisfaction.

## Supporting information

S1 Table After-care instant patient satisfaction survey questionnaire. (DOCX)

S2 Table Linear contrasts comparing JES score quartiles. (DOCX)
