# Supplementary material for: Association between emergency physician self-reported empathy and patient satisfaction
Source: PLoS One. 2018 Sep 13;13(9):e0204113. doi: 10.1371/journal.pone.0204113 (PMC6136813; doi:10.1371/journal.pone.0204113)
Supplement: S1 Table — (DOCX) [file pone.0204113.s001.docx]

Supplemental Table 1 – After-care Instant Patient Satisfaction Survey Questionnaire:

Patient Name (if you are willing to provide) ____________

Who completed the survey: Patient, Family Member, Legal Guardian, Friend, Other?

1. Who was the Registered Nurse that treated you today?

Answer: ________

1. How satisfied were you with the level of care shown by the nursing staff?

Very dissatisfied Dissatisfied Neither satisfied nor dissatisfied Satisfied Very Satisfied

1. Who was the attending doctor that treated you today?

Answer: _________

1. How satisfied were you with the level of care shown by your attending doctor?

Very dissatisfied Dissatisfied Neither satisfied nor dissatisfied Satisfied Very Satisfied

1. Who was the resident doctor that treated you today?

Answer: _________

1. How satisfied were you with the level of care shown by your resident doctor?

Very dissatisfied Dissatisfied Neither satisfied nor dissatisfied Satisfied Very Satisfied

1. Who was the Physician Assistant, or Nurse Practitioner that treated you today?

Answer: _________

1. How satisfied were you with the level of care shown by your Physician Assistant, or Nurse Practitioner?

Very dissatisfied Dissatisfied Neither satisfied nor dissatisfied Satisfied Very Satisfied

1. How often was your pain well controlled?

Do not have pain Rarely Sometime Usually Always

1. Overall, how satisfied were you with your visit today? (Scale of 1-10, 1: very dissatisfied, 10 very satisfied)

Answer: ___________
