# Supplementary material for: Association between emergency physician self-reported empathy and patient satisfaction
Source: PLoS One. 2018 Sep 13;13(9):e0204113. doi: 10.1371/journal.pone.0204113 (PMC6136813; doi:10.1371/journal.pone.0204113)
Supplement: S2 Table — (DOCX) [file pone.0204113.s002.docx]

Supplemental Table 2. Linear contrasts comparing JES score quartiles

|  | Contrasts | RR | LCL | UCL |  |
| --- | --- | --- | --- | --- | --- |
| JES quartile | 1 vs 2 | 0.89 | 0.79 | 1.00 |  |
|  | 1 vs 3 | 0.86 | 0.76 | 0.96 |  |
|  | 1 vs 4 | 0.89 | 0.80 | 1.01 |  |
|  | 2 vs 3 | 0.96 | 0.87 | 1.08 | This is where the ceiling effect is starting to become more evident |
|  | 2 vs 4 | 1.01 | 0.90 | 1.12 |  |
|  | 3 vs 4 | 1.04 | 0.94 | 1.16 |  |
| JES-Jefferson Empathy Scale | | |  |  |  |
| RR-Risk Ratio; LCL-95% lower confidence limit; UCL-95% upper confidence limit | | | | | |
| Quartile 1 ranged between 80 and 107 | | | |  |  |
| Quartile 2 ranged between 107 and 115 | | | |  |  |
| Quartile 3 ranged between 115 and 125 | | | |  |  |
| Quartile 4 ranged between 125 and 134 | | | |  |  |
